# Redescription of two species of *Neoperla* Needham, 1905 (Plecoptera, Perlidae) and new distribution records of *Neoperlamnong* Stark, 1987 in China

**DOI:** 10.3897/BDJ.12.e127089

**Published:** 2024-06-27

**Authors:** Liang-Liang Zeng, Qing-Bo Huo, Yu-Zhou Du

**Affiliations:** 1 College of Plant Protection & Institute of Applied Entomology, Yangzhou University, Yangzhou 225009, China, Yangzhou, China College of Plant Protection & Institute of Applied Entomology, Yangzhou University, Yangzhou 225009, China Yangzhou China; 2 Joint International Research Laboratory of Agriculture and Agri-Product Safety, the Ministry of Education, Yangzhou University, Yangzhou 225009, China, Yangzhou, China Joint International Research Laboratory of Agriculture and Agri-Product Safety, the Ministry of Education, Yangzhou University, Yangzhou 225009, China Yangzhou China

**Keywords:** Stonefly, *Neoperlabituberculata*, *Neoperladashahena*, *Neoperlamnong*

## Abstract

**Background:**

Two species of *Neoperla* from Guizhou Province, China, *N.bituberculata* Du, 2000 and *N.dashahena* Du, 2005 were described with brief morphological descriptions available only in Chinese and original illustrations being somewhat blurry. Recently, we examined type material of these two species and re-described them with clear colour pictures for the first time.

**New information:**

In this paper, detailed English descriptions and colour pictures of *Neoperlabituberculata* and *N.dashahena* are provided for the first time. The type locality of *N.mnong* Stark is from Vietnam and its geographical distribution is also discussed. Additionally, we also recorded the distribution of *N.mnong* Stark, 1987 in Guizhou, Hunan and Jiangxi Provinces of China for the first time and provided a geographical distribution map of this species.

## Introduction

*Neoperla* Needham, 1905 is the most species-rich genus in Perlidae, with at least 372 known species (Zwick 2023, DeWalt et al. 2024). It is mainly distributed in eastern North America, Tropical and Temperate Asia and central Africa (Illies 1966, Zwick 1973, Stark and Gaufin 1976, DeWalt et al. 2024). The morphology of the highly differentiated penis provides the most important diagnostic characteristics for species identification in *Neoperla* (Zwick 1983, Sivec et al. 1988). Guizhou is bordered by Sichuan Province and Chongqing Municipality to the north, Hunan Province to the east, Guangxi Zhuang Autonomous Region to the south and Yunnan Province to the west. Presently, there are eleven *Neoperla* species known to occur in Guizhou. However, the morphological characteristics of some of these species, especially the original drawing of the penis, are not detailed enough and the chronic lack of descriptions makes identification difficult. It is necessary to reorganise and re-describe the preserved *Neoperla* specimens from Guizhou.

Recently, we found and examined the type material of *N.bituberculata* (Du 2000) and *N.dashahena* (Du and Wang 2005) when examining the *Neoperla* materials collected from Guizhou. It is re-described and for the first time provided with clear colour pictures and morphological characteristics are compared with closely-related species. In addition, we provide the first record of *N.mnong* in Guizhou, Hunan and Jiangxi, China. The type locality of this species is Vietnam (Di Linh), which was previously reported only in Guangdong and Guangxi, China. In this paper, the distribution maps of these species are updated and the zoogeography is discussed.

## Materials and methods

Specimens were collected by light trap and Malaise trap. All the materials are preserved in 75% ethanol. Photographs were taken with the KEYENCE VHX-5000 system and subsequently optimised in Adobe Photoshop CS6. All the specimens are deposited in the Insect Collection of Yangzhou University (ICYZU), Jiangsu Province, China. Terminology follows that of Zwick (2023).

## Taxon treatments

### Neoperla dashahena

Du, 2005

5B52B6D2-2250-5E1B-AB4A-52194FC7B8DE

Neoperla dashahena Du, 2005: 51.

#### Materials

- **Type status:** Holotype. **Occurrence:** recordedBy: Du Yu-Zhou; individualCount: 1; sex: 1 male; **Taxon:** scientificNameID: *Neoperladashahena* Du, 2005; kingdom: Animalia; phylum: Arthropoda; class: Insecta; order: Plecoptera; family: Perlidae; genus: *Neoperla*; **Location:** country: China; countryCode: CN; stateProvince: Guizhou Province; county: Daozhen; locality: Dasha River National Nature Reserve, Xiannvdong; **Identification:** identifiedBy: Zeng Liang-liang, Du Yu-Zhou, Huo Qing-Bo; **Event:** year: 2004; month: 5; day: 22-29; **Record Level:** language: en; institutionCode: ICYZU; basisOfRecord: PreservedSpecimen
- **Type status:** Paratype. **Occurrence:** recordedBy: Du Yu-Zhou; sex: 3 males; lifeStage: adult; **Taxon:** scientificNameID: *Neoperladashahena* Du, 2005; kingdom: Animalia; phylum: Arthropoda; class: Insecta; order: Plecoptera; family: Perlidae; genus: *Neoperla*; **Location:** country: China; countryCode: CN; stateProvince: Guizhou Province; county: Daozhen; locality: Dasha River National Nature Reserve, Xiannvdong; **Identification:** identifiedBy: Zeng Liang-liang, Du Yu-Zhou, Huo Qing-Bo; **Event:** year: 2004; month: 5; day: 22-29; **Record Level:** language: en; institutionCode: ICYZU; basisOfRecord: PreservedSpecimen

#### Description

**Figure 1.**
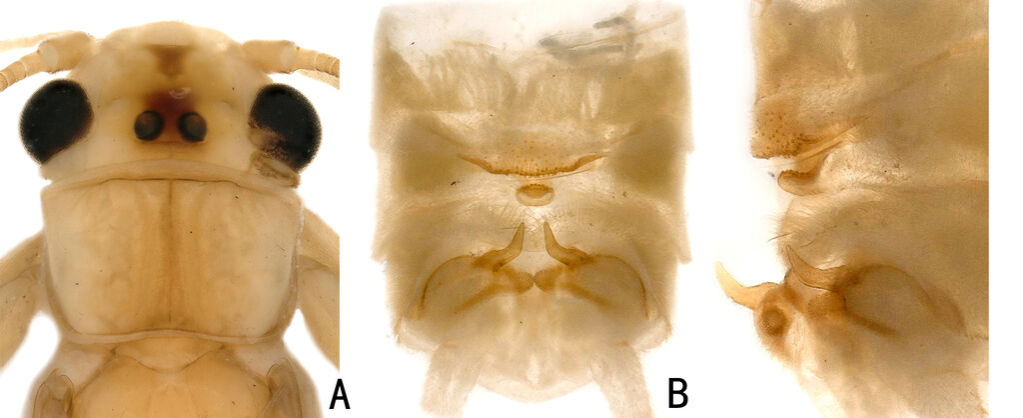
*Neoperladashahena* Du, 2005 from Guizhou, male. **A** head and pronotum, dorsal view; **B** abdomen, dorsal view; **C** abdomen, lateral view.

**Adult habitus:** General body colour brown. The head is pale yellow with two ocelli. The ocelli are black, surrounded by a quadrate brown area. An inverted brown triangle is present centrally on the frons (Fig. 1A); Pronotum is light brown and trapezoidal in shape; Abdominal segments and cerci pale brown.

**Male: **The middle posterior part of tergite 7 has square or quadrate areas that are slightly raised, the posterior margin of which is sclerotised and has a small sensilla basiconica patch. As specimens of this species have been preserved in alcohol for nearly 20 years, lateral sclerotised areas give the impression of a broad, shallow arc. Tergite 8 bears a recurved tongue-like process with small spines at the distal margin. Hemitergal processes of tergite 10 up-curved, extending backwards to the central process of tergite 9, tip rounded. (Fig. 1B-C).

**Figure 2.**
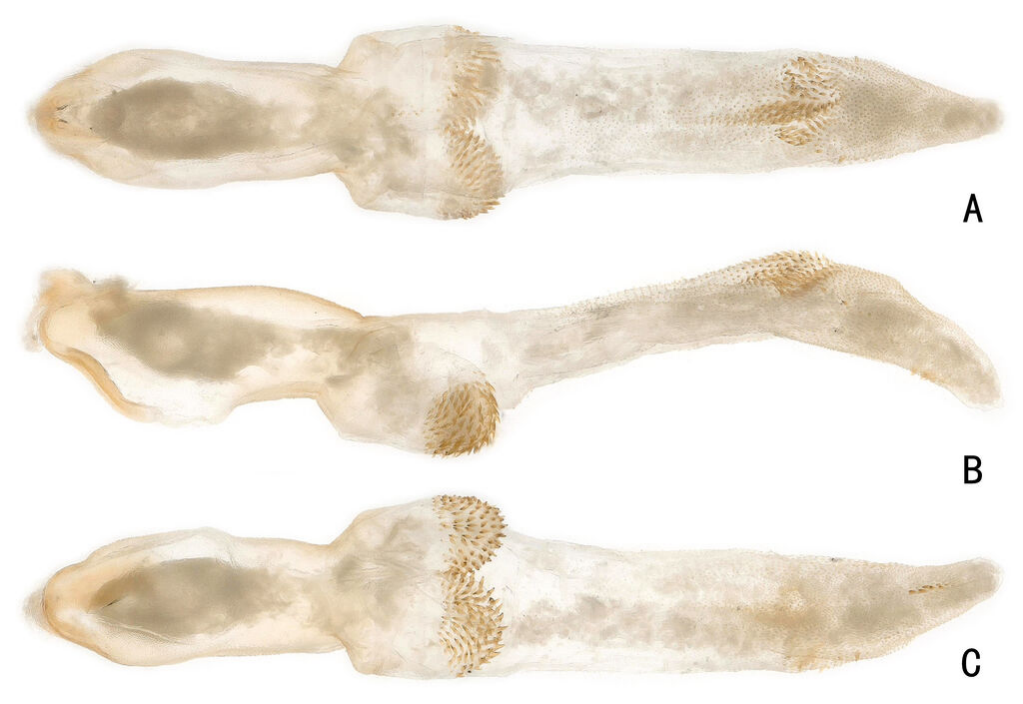
*Neoperladashahena* Du, 2005 from Guizhou, male. **A** penis base and everted endophallus, dorsal view; **B** penis base and everted endophallus, lateral view; **C** penis base and everted endophallus, ventral view.

**Figure 3.**
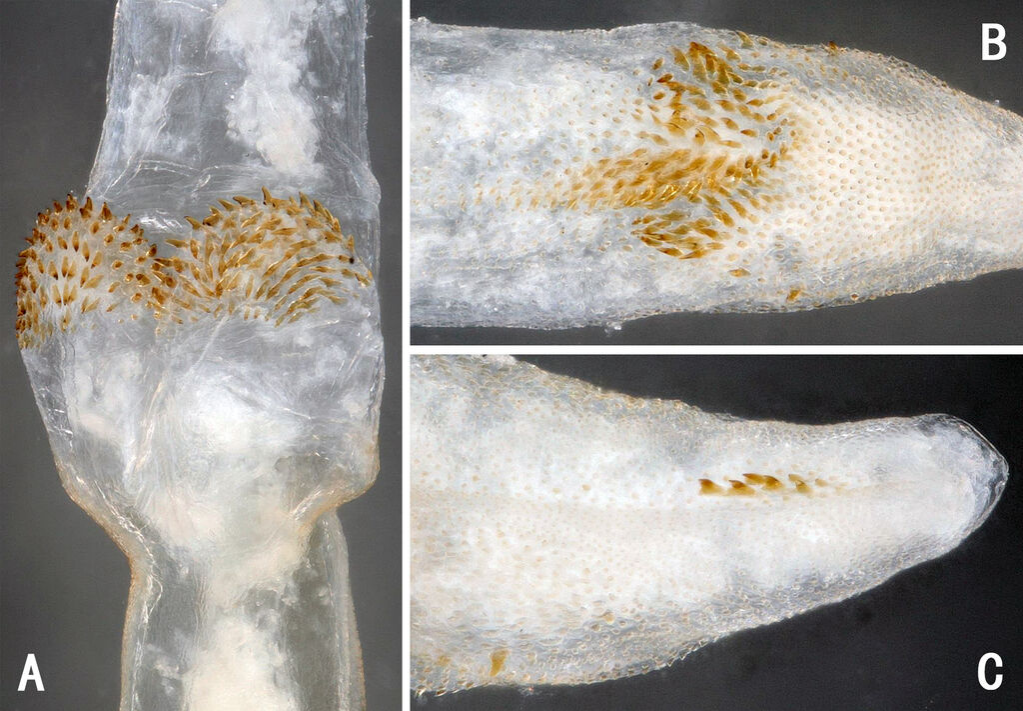
*Neoperladashahena* Du, 2005 from Guizhou, male. **A** ventral surface of anti-apical endophallus, ventral view; **B** apical half of everted endophallus, dorsal view; **C** apex of everted endophallus, ventral view.

The medial pair of spinule-covered projections appear to be part of the penis base. The endophallus starts distal of these lobes. The connected distal patch of spinules is located anti-apically on the endophallus. (Fig. 2B-C and Fig. 3A). Larger spinule patch on the dorsal surface of the middle part of the everted endophallus (Fig. 2A and Fig. 3B), with a row of small spines on the ventral surface of the proximal endophallus (Fig. 2C and Fig. 3C).

#### Distribution

China (Guizhou).

#### Taxon discussion

*Neoperladashahena* was originally assigned to the montivaga species group (Zwick 1983, Zwick 1986) with the penis having incomplete sclerotisation in the ventral aspect. Zwick (2023) recently made this grouping obsolete and erected two subgenera, Neoperla (Borneella) and Neoperla (Formosita) Klapálek.

Due to the presence of paired, ventral spiny lobes at the apex of the penis base, we now assign *N.dashahena* to the *N.*(*Formosita*) *lushana*-group. The penis of *N.dashahena* is most similar to *Neoperlalatamaculata* Du, 2005 and *Neoperlayaoshana* Li, Wang & Lu, 2011. In *N.dashahena*, a larger spinule patch exists on the dorsal surface of the everted endophallus, with a row of small spines on the ventral surface of the endophallus. In *N.latamaculata*, the dorsal surface of the everted endophallus has a "V"-shaped spinules patch. In *N.yaoshana*, the apical half of the everted endophallus bears a field of fine dorsal and ventral spinules separated by a lateral membranous area.

### Neoperla bituberculata

Du, 2000

0A250BFC-602F-51E3-8992-7DEA47754D06

Neoperla bituberculata Du, 2000: 1.

#### Materials

- **Type status:** Holotype. **Occurrence:** recordedBy: Du Yu-Zhou; individualCount: 1; sex: 1 male; lifeStage: adult; **Taxon:** scientificName: *Neoperlabituberculata* Du, 2000; kingdom: Animalia; phylum: Arthropoda; class: Insecta; order: Plecoptera; family: Perlidae; genus: *Neoperla*; **Location:** country: China; countryCode: CN; stateProvince: Guizhou Province; county: Libo; locality: Maolan National Nature Reserve, Sancha River.; **Identification:** identifiedBy: Du Yu-Zhou, Huo Qing-Bo, Zeng Liang-Liang; **Event:** year: 1994; month: 7; day: 8; **Record Level:** language: en; institutionID: ICYZU
- **Type status:** Paratype. **Occurrence:** recordedBy: Du Yu-Zhou; individualCount: 1; sex: 1 male; lifeStage: adult; **Taxon:** scientificName: *Neoperlabituberculata* Du, 2000; kingdom: Animalia; phylum: Arthropoda; class: Insecta; order: Plecoptera; family: Perlidae; genus: *Neoperla*; **Location:** country: China; countryCode: CN; stateProvince: Guizhou Province; county: Libo; locality: Maolan National Nature Reserve, Sancha River.; **Identification:** identifiedBy: Du Yu-Zhou, Huo Qing-Bo, Zeng Liang-Liang; **Event:** year: 1994; month: 7; day: 8; **Record Level:** language: en; institutionID: ICYZU

#### Description

**Figure 4.**
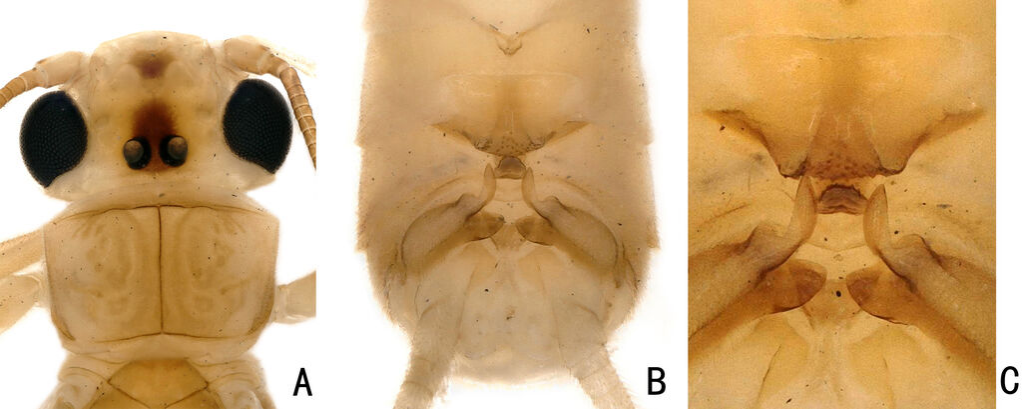
*Neoperlabituberculata* Du, 2005 from Guizhou, male. **A** head and pronotum, dorsal view; **B** abdomen, dorsal view; **C** abdomen, dorsal view.

**Adult habitus:** General body colour brown. Head mostly yellowish-brown, with a black marking covering an ocellar triangle; Compound eyes black; Pronotum disc brown, mid-line darker, margins pale (Fig. 4A).

**Male: **The anterior edge of tergite 7 is concave in the middle and forms a "Y"-shape with one projecting sclerite. In the posterior part of the tergite, there is a raised process that slightly bifurcates to form a median ridge covered by many small sensilla basiconica. Tergite 8 has a tongue-shaped upcurved process, with many sensilla basiconica at the distal margin. Hemitergal processes of tergite 10 sclerotiszed and finger-like, a subapical protrusion is present (Fig. 4B–C).

**Figure 5.**
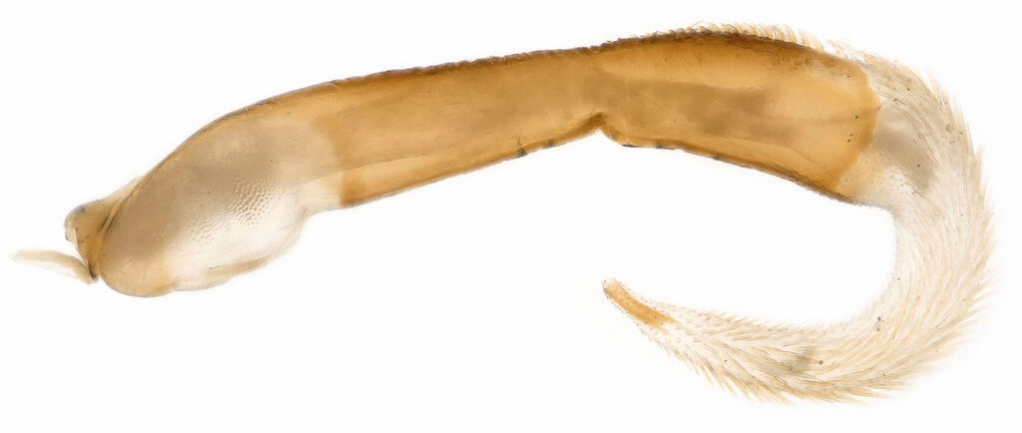
*Neoperlabituberculata* Du, 2005 from Guizhou, male. Penis base and everted endophallus, lateral view.

Penis base well sclerotised and its dorsal surface near the tip has small spines (Fig. 5). The everted endophallus curves ventrally, with a tube-like inner sclerite apically. The entire endophallus is densely covered with slender golden-brown spines.

#### Distribution

China (Guizhou).

#### Taxon discussion

This species is similar to *Neoperla* *infuscata* Wu, 1935, but their everted endophallus are quite different. In *N.bituberculata*, there are many golden-brown spines on the endophallus, while in *N.infuscata*, the endophallus bears only a few small spines. In addition, the tergite 7 of the *N.bituberculata* has two small lobes, which is also used for the etymology for this species.

### Neoperla mnong

Stark, 1987

868C225F-19A0-51E4-BB1A-69D9236D599C

Javanita costalis Navás, 1932: 925. Secondary homonym of *Formosinacostalis* Klapálek (Zwick 1988)
Neoperla mnong Stark, 1987: 48. Holotype ♂ (California Academy of Sciences). Di Linh, Vietnam
Neoperla angustilobata Zwick, 1988: 404. Holotype ♀ (Muséum National d’Histoire Naturelle), New Synonymy

#### Materials

- **Type status:** Other material. **Occurrence:** recordedBy: Du Yu-Zhou; individualCount: 1; sex: 1 male; lifeStage: adult; occurrenceID: C53BD24C-67C4-52EC-B0BA-9AE9AAFEE10D; **Taxon:** scientificName: *Neoperlamnong* Stark, 1987; kingdom: Animalia; phylum: Arthropoda; class: Insecta; order: Plecoptera; family: Perlidae; genus: *Neoperla*; **Location:** country: China; stateProvince: Guizhou Province; county: Leishan; locality: Danjiang Village; **Identification:** identifiedBy: Du Yu-Zhou, Huo Qing-Bo, Zeng Liang-Liang; **Event:** year: 2005; month: 6; day: 4; **Record Level:** language: en; institutionCode: ICYZU
- **Type status:** Other material. **Occurrence:** recordedBy: Zeng Liang-Liang; individualCount: 3; sex: 3 males; lifeStage: adult; occurrenceID: 63B63C98-FCD6-58E1-A968-2733B2D92A93; **Taxon:** scientificName: *Neoperlamnong* Stark, 1987; kingdom: Animalia; phylum: Arthropoda; class: Insecta; order: Plecoptera; family: Perlidae; genus: *Neoperla*; **Location:** country: China; stateProvince: Guangdong Province; county: Shixing; locality: Chebaling National Nature Reserve; **Identification:** identifiedBy: Du Yu-Zhou, Huo Qing-Bo, Zeng Liang-Liang; **Event:** year: 2020; month: 8; day: 23; **Record Level:** language: en; institutionCode: ICYZU
- **Type status:** Other material. **Occurrence:** recordedBy: Zeng Liang-Liang; individualCount: 2; sex: 2 males; lifeStage: adult; occurrenceID: 2820260E-0D2A-58EC-9D74-D8D11700AF60; **Taxon:** scientificName: *Neoperlamnong* Stark, 1987; kingdom: Animalia; phylum: Arthropoda; class: Insecta; order: Plecoptera; family: Perlidae; genus: *Neoperla*; **Location:** country: China; stateProvince: Jiangxi Province; county: Longnan; locality: Jiulianshan National Nature Reserve; **Identification:** identifiedBy: Du Yu-Zhou, Huo Qing-Bo, Zeng Liang-Liang; **Event:** year: 2020; month: 7; day: 7-2; **Record Level:** language: en; institutionCode: ICYZU
- **Type status:** Other material. **Occurrence:** recordedBy: Zeng Liang-Liang; individualCount: 2; sex: 2 males; lifeStage: adult; occurrenceID: B39AD1D6-AFDC-57A3-8EE3-E16F30D86640; **Taxon:** scientificName: *Neoperlamnong* Stark, 1987; kingdom: Animalia; phylum: Arthropoda; class: Insecta; order: Plecoptera; family: Perlidae; genus: *Neoperla*; **Location:** country: China; stateProvince: Hunan Province; county: Huitong; locality: Yingzuijie National Nature Reserve; **Identification:** identifiedBy: Du Yu-Zhou, Huo Qing-Bo, Zeng Liang-Liang; **Event:** year: 2023; month: 7; day: 14; **Record Level:** language: en; institutionCode: ICYZU

#### Distribution

 China (Guizhou, Hunan, Jiangxi, Guangdong, Guangxi); Vietnam; Thailand.

#### Taxon discussion

Stark and Sivec (2008) mentioned that *N.mnong* was first described as *Javanitacostalis* (Navas 1932) from a female specimen and later described as a new species from a male specimen (Stark 1987). Due to their similar penis, *N.mnong*, *N.han* Stark, 1987, *N.furcostyla* Li & Qin, 2013, *N.forcipata* Yang and Yang, 1992 and *N.yao* Stark, 1987 were classified into the *diehli* subgroup (Wang et al. 2013). Subsequently, these species were assigned to the *N.* (*Formosita*) *diehli* complex (group) (Zwick 2023). It needs to be corrected as the penis of *N.mnong* was misidentified as *N.dao* Stark & Sivec, 2008 in Zwick (2023), on page 116, figure 67. The *diehli* complex (group) is primarily distributed in low-latitude regions of East Asia to South Asia, including southern China (Guangdong, Guangxi, Yunnan, Hainan etc.) to areas spanning Vietnam, Thailand and Indonesia.

**Figure 6.**
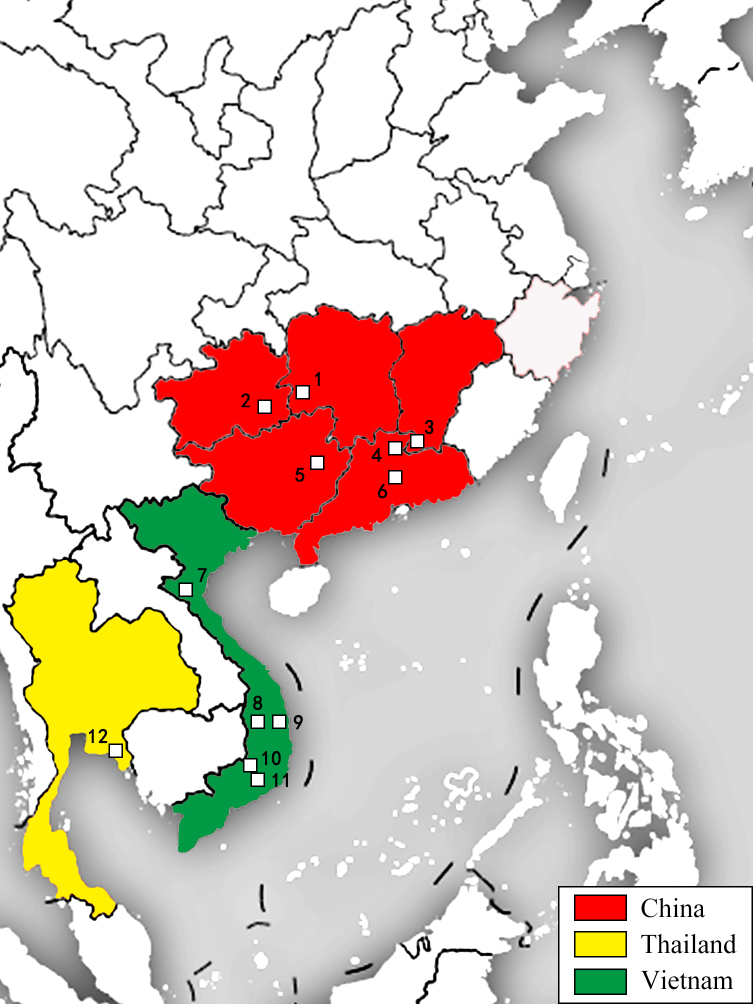
The distribution of *Neoperlamnong* Stark, 1987. The white squares indicate the collection places. China: 1. Hunan, Huitong; 2. Guizhou, Leishan; 3. Jiangxi, Longnan; 4. Guangdong, Shixing; 5. Guangxi, Jinxiu; 6. Guangdong, Conghua; Vietnam: 7. Con Cuong; 8. Pleiku; 9. An Khe; 10. Dak Son; 11. Di Linh; 12. Thailand, Chanthaburia.

**Figure 7.**
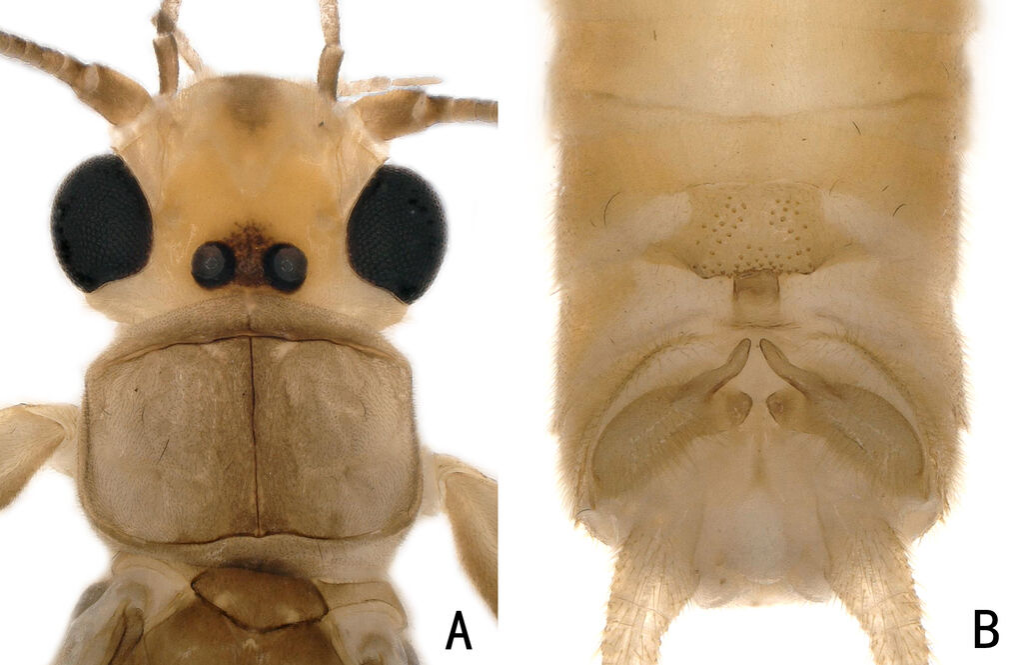
*Neoperlamnong* Stark, 1987, male. **A** head and pronotum, dorsal view; **B** abdomen, dorsal view.

**Figure 8.**
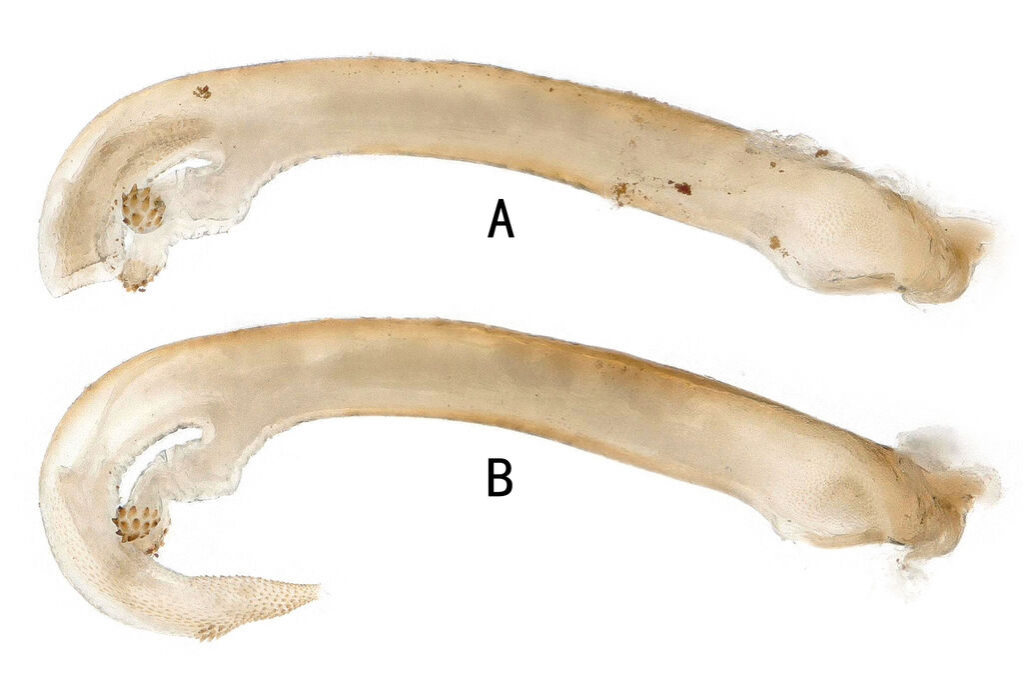
*Neoperlamnong* Stark, 1987, male. **A** penis with partly everted endophallus, lateral view; **B** penis base and everted endophallus, lateral view.

This article records for the first time the distribution of this species in Guizhou, Hunan and Jiangxi Provinces of China, covering most of the southern region of China to Vietnam, indicating that it is a species that is widely distributed in coastal areas of Southeast Asia (Fig. 6). In China, this species is primarily concentrated in the Nanling Mountains, but records from Guizhou and Hunan suggest that it may also occur in adjacent inland provinces to the west (or north) of the Nanling Mountains. In the future, more widespread species or new distribution records of the diehli complex (group) may be reported. We provide additional illustrations (Figs 7, 8) to aid in the recognition of this species.

## Supplementary Material

XML Treatment for Neoperla dashahena

XML Treatment for Neoperla bituberculata

XML Treatment for Neoperla mnong
